# Bis(1,10-phenanthroline-κ^2^ *N*,*N*′)(sulfato-κ^2^ *O*,*O*′)nickel(II) propane-1,3-diol solvate

**DOI:** 10.1107/S1600536810020210

**Published:** 2010-06-05

**Authors:** Chao Ni, Kai-Long Zhong, Jiang-Dong Cui

**Affiliations:** a Department of Applied Chemistry, Nanjing College of Chemical Technology, Nanjing 210048, People’s Republic of China

## Abstract

In the structure of the title compound, [Ni(SO_4_)(C_12_H_8_N_2_)_2_]·C_3_H_8_O_2_, the Ni^II^ ion (site symmetry 2) is six-coordinated in a distorted octa­hedral manner by four N atoms from two chelating 1,10-phenanthroline (phen) ligands and two O atoms from a bidentate sulfate ligand (2 symmetry). The dihedral angle between the two chelating NCCN groups is 80.9 (1)°. The central C atom of the propane-1,3-diol solvent mol­ecule is likewise located on a twofold rotation axis. In the crystal structure, the [Ni(SO_4_)(C_12_H_8_N_2_)_2_] and C_3_H_8_O_2_ entities are connected through inter­molecular O—H⋯O hydrogen bonding.

## Related literature

For the isotypic Zn and Co structures, see: Cui *et al.* (2010 ▶) and Zhong (2010 ▶), respectively. For the ethane-1,2-diol solvate of the title complex, see: Zhong *et al.* (2009 ▶). For background to coordination polymers constructed from N-containing ligands, see: Zhang *et al.* (1999 ▶); Blake *et al.* (2007 ▶); Wang *et al.* (2007 ▶).
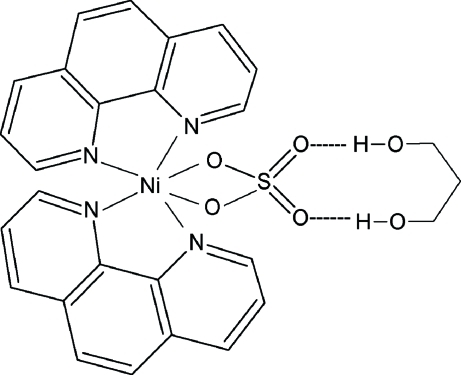


## Experimental

### 

#### Crystal data

[Ni(SO_4_)(C_12_H_8_N_2_)_2_]·C_3_H_8_O_2_
*M* *_r_* = 591.26
Monoclinic, 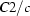

*a* = 18.243 (4) Å
*b* = 12.440 (3) Å
*c* = 13.180 (3) Å
β = 121.58 (3)°
*V* = 2548.2 (13) Å^3^
*Z* = 4
Mo *K*α radiation
μ = 0.90 mm^−1^
*T* = 223 K
0.55 × 0.50 × 0.40 mm

#### Data collection

Rigaku Mercury CCD diffractometer
Absorption correction: multi-scan (*REQAB*: Jacobson, 1998 ▶) *T*_min_ = 0.750, *T*_max_ = 1.000
7078 measured reflections
2877 independent reflections
2630 reflections with *I* > 2σ(*I*)
*R*_int_ = 0.017

#### Refinement

*R*[*F* ^2^ > 2σ(*F* ^2^)] = 0.034
*wR*(*F* ^2^) = 0.093
*S* = 1.05
2877 reflections
179 parameters
H-atom parameters constrained
Δρ_max_ = 0.79 e Å^−3^
Δρ_min_ = −0.42 e Å^−3^

### 

Data collection: *CrystalClear* (Rigaku, 2007 ▶); cell refinement: *CrystalClear*; data reduction: *CrystalClear*; program(s) used to solve structure: *SHELXS97* (Sheldrick, 2008 ▶); program(s) used to refine structure: *SHELXL97* (Sheldrick, 2008 ▶); molecular graphics: *XP* in *SHELXTL* (Sheldrick, 2008 ▶); software used to prepare material for publication: *SHELXTL*.

## Supplementary Material

Crystal structure: contains datablocks global, I. DOI: 10.1107/S1600536810020210/wm2347sup1.cif

Structure factors: contains datablocks I. DOI: 10.1107/S1600536810020210/wm2347Isup2.hkl

Additional supplementary materials:  crystallographic information; 3D view; checkCIF report

## Figures and Tables

**Table:** 

Ni1—N2	2.0775 (16)
Ni1—N1	2.0802 (16)
Ni1—O1	2.1127 (14)
S1—O2	1.4559 (14)
S1—O1	1.4950 (14)

**Table:** 

N2—Ni1—N1	80.05 (6)
O1^i^—Ni1—O1	67.73 (7)

Symmetry code: (i) 
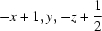
.

**Table 2:** Hydrogen-bond geometry (Å, °)

*D*—H⋯*A*	*D*—H	H⋯*A*	*D*⋯*A*	*D*—H⋯*A*
O3—H3⋯O2	0.82	1.92	2.743 (2)	179

**Fig. 1.**
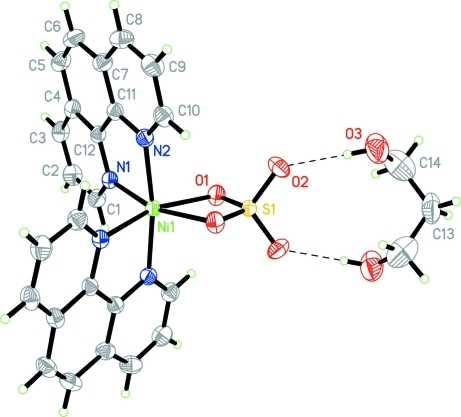
The molecular structure of the title compound showing the atom-numbering scheme. Displacement ellipsoids are drawn at the 50% probability level. The dashed lines represent O—H···O interactions. Unlabeled atoms are related to the labelled atoms by the symmetry operator (-x+1, y, -z+1/2).

## supplementary crystallographic information

### Comment

Many N containing ligands, such as 1,10-phenanthroline, 4,4'-bipyridine and 2,2'-bipyridine have been widely applied in constructing coordination polymers as auxiliary ligands (Zhang *et al.*, 1999; Blake *et al.*, 2007; Wang *et al.*, 2007). The title nickel compound, [NiSO_4_(C_12_H_8_N_2_)_2_]^.^C_3_H_8_O_2_, (I), was obtained unintentionally during an attempt to synthesize coordination polymers of Ni^II^ with 1,10-phenanthroline as second ligand *via* a solvothermal reaction. (I) is isotypic with the recently reported cobalt(II) and zinc(II) structures [Zhong 2010, (II); Cui *et al.*, 2010, (III)].

The metal complex and solvent entities of (I) are held together by two intermolecular O—H···O hydrogen bonds including the uncoordinated O atoms of the sulfate group (Fig. 1). In the complex molecule, the Ni^II^ atom is six-coordinated in a distorted octahedral manner by four N atoms from two chelating 1,10-phenanthroline (phen) ligands and two O atoms from a bidentate-chelating sulfate anion. The Ni—O bond length [2.1127 (14) Å], the O—Ni—O bite angle [67.73 (7)°], the Ni—N bond lengths [2.0775 (16) and 2.0802 (16) Å], and the N—Ni—N bite angle [80.05 (6)°] are in good agreement with those of the observed in the ethane-1,2-diol solvate [NiSO_4_(C_12_H_8_N_2_)_2_]^.^C_2_H_6_O_2_, (IV), [2.1077 (16) Å, 67.58 (8)°, 2.0774 (18), 2.0805 (15)Å and 79.99 (7)°, respectively; Zhong *et al.*, 2009]. The two chelating NCCN groups have a dihedral angle of 80.9 (1)°, which is much larger than that found in the structure of (IV), 71.0°. The Ni^II^, the S  and the central C atom of the propane-1,3-diol solvent molecule lie on a twofold rotation axis. Selected bond lengths and angles are compiled in Table 1 and intermolecular hydrogen bonding in Table 2, respectively.

In the title complex, the geometry of the phen and sulfate ligands is in good agreement with those of the previously reported metal complexes, (II), (III) and (IV). The phen ligands are all planar with the largest deviation of atoms from their mean plane less than 0.03 Å. The bond distances and angles in phen [1.353 (3)-1.436 (2) Å and 116.97 (17)-123.95 (17)° Å, respectivly] are all normal. The S—O distances within the sulfate ligands, 1.4559 (14) Å for and 1.4950 (14) Å are also similar to those observed for (II), (III) and (VI).

### Experimental

0.2 mmol phen, 0.1 mmol melamine, 0.1 mmol NiSO_4_^.^7H_2_O, 2.0 ml propane-1,3-diol and 1.0 ml water were mixed and placed in a thick Pyrex tube, which was sealed and heated to 413 K for 96 h. Blue block-shaped crystals of (I) were obtained after the reaction time.

### Refinement

The H atoms of phen were positioned geometrically and allowed to ride on their parent atoms, with C—H distances of 0.93 Å and *U*_iso_(H) = 1.2*U*_eq_(C). The other H atoms were placed in geometrically idealized positions and refined as riding atoms, with C—H = 0.97 Å and O—H = 0.82 Å; *U*_iso_(H) = 1.2*U*_eq_(C) and 1.5*U*_eq_(O).

### Crystal data

**Table:**

[Ni(SO_4_)(C_12_H_8_N_2_)_2_]·C_3_H_8_O_2_	*F*(000) = 1224
*M**_r_* = 591.26	*D*_x_ = 1.541 Mg m^−^^3^
Monoclinic, *C*2/*c*	Mo *K*α radiation, λ = 0.71073 Å
Hall symbol: -C 2yc	Cell parameters from 6113 reflections
*a* = 18.243 (4) Å	θ = 3.2–27.5°
*b* = 12.440 (3) Å	µ = 0.90 mm^−^^1^
*c* = 13.180 (3) Å	*T* = 223 K
β = 121.58 (3)°	Block, blue
*V* = 2548.2 (13) Å^3^	0.55 × 0.50 × 0.40 mm
*Z* = 4	

### Data collection

**Table:**

Rigaku Mercury CCD diffractometer	2877 independent reflections
Radiation source: fine-focus sealed tube	2630 reflections with *I* > 2σ(*I*)
Graphite Monochromator	*R*_int_ = 0.017
Detector resolution: 28.5714 pixels mm^-1^	θ_max_ = 27.5°, θ_min_ = 3.2°
ω scans	*h* = −23→23
Absorption correction: multi-scan (REQAB: Jacobson, 1998)	*k* = −16→13
*T*_min_ = 0.750, *T*_max_ = 1.000	*l* = −16→17
7078 measured reflections	

### Refinement

**Table:**

Refinement on *F*^2^	Secondary atom site location: difference Fourier map
Least-squares matrix: full	Hydrogen site location: inferred from neighbouring sites
*R*[*F*^2^ > 2σ(*F*^2^)] = 0.034	H-atom parameters constrained
*wR*(*F*^2^) = 0.093	*w* = 1/[σ^2^(*F*_o_^2^) + (0.0574*P*)^2^ + 1.7246*P*] where *P* = (*F*_o_^2^ + 2*F*_c_^2^)/3
*S* = 1.05	(Δ/σ)_max_ < 0.001
2877 reflections	Δρ_max_ = 0.79 e Å^−^^3^
179 parameters	Δρ_min_ = −0.42 e Å^−^^3^
0 restraints	Extinction correction: *SHELXL97* (Sheldrick, 2008), Fc^*^=kFc[1+0.001xFc^2^λ^3^/sin(2θ)]^-1/4^
Primary atom site location: structure-invariant direct methods	Extinction coefficient: 0.0115 (8)

### Special details

**Table:**

Geometry. All esds (except the esd in the dihedral angle between two l.s. planes) are estimated using the full covariance matrix. The cell esds are taken into account individually in the estimation of esds in distances, angles and torsion angles; correlations between esds in cell parameters are only used when they are defined by crystal symmetry. An approximate (isotropic) treatment of cell esds is used for estimating esds involving l.s. planes.
Refinement. Refinement of *F*^2^ against ALL reflections. The weighted *R*-factor wR and goodness of fit *S* are based on *F*^2^, conventional *R*-factors *R* are based on *F*, with *F* set to zero for negative *F*^2^. The threshold expression of *F*^2^ > σ(*F*^2^) is used only for calculating *R*-factors(gt) etc. and is not relevant to the choice of reflections for refinement. *R*-factors based on *F*^2^ are statistically about twice as large as those based on *F*, and *R*- factors based on ALL data will be even larger.

### Fractional atomic coordinates and isotropic or equivalent isotropic displacement parameters (Å^2^)

**Table:**

	*x*	*y*	*z*	*U*_iso_*/*U*_eq_	
Ni1	0.5000	0.31623 (2)	0.2500	0.02219 (13)	
S1	0.5000	0.53132 (5)	0.2500	0.02350 (16)	
O1	0.44210 (8)	0.45725 (10)	0.15044 (11)	0.0300 (3)	
O2	0.44999 (10)	0.59777 (13)	0.28297 (15)	0.0422 (4)	
N1	0.40871 (10)	0.20994 (12)	0.12675 (13)	0.0245 (3)	
N2	0.41995 (10)	0.30246 (12)	0.31754 (14)	0.0252 (3)	
C2	0.33170 (14)	0.10453 (17)	−0.05175 (18)	0.0351 (4)	
H2A	0.3282	0.0788	−0.1204	0.042*	
C6	0.22391 (12)	0.13600 (17)	0.21208 (18)	0.0345 (4)	
H6A	0.1843	0.1178	0.2334	0.041*	
C7	0.29358 (12)	0.20714 (16)	0.28623 (17)	0.0289 (4)	
C9	0.37020 (14)	0.32691 (16)	0.45183 (18)	0.0354 (4)	
H9A	0.3774	0.3602	0.5197	0.043*	
C5	0.21523 (12)	0.09508 (16)	0.11125 (18)	0.0338 (4)	
H5A	0.1685	0.0509	0.0630	0.041*	
C4	0.27619 (11)	0.11813 (14)	0.07706 (16)	0.0275 (4)	
C10	0.42736 (12)	0.34793 (16)	0.41402 (17)	0.0303 (4)	
H10A	0.4725	0.3956	0.4580	0.036*	
C8	0.30380 (13)	0.25725 (17)	0.38851 (17)	0.0356 (4)	
H8A	0.2653	0.2429	0.4129	0.043*	
C1	0.40052 (13)	0.17061 (15)	0.02734 (17)	0.0305 (4)	
H1A	0.4422	0.1877	0.0096	0.037*	
C11	0.35423 (11)	0.23219 (13)	0.25508 (15)	0.0239 (3)	
C3	0.26998 (13)	0.07832 (15)	−0.02736 (17)	0.0325 (4)	
H3A	0.2241	0.0345	−0.0792	0.039*	
C12	0.34677 (11)	0.18502 (13)	0.15061 (16)	0.0233 (3)	
C14	0.4235 (2)	0.8716 (3)	0.2220 (3)	0.0797 (10)	
H14A	0.3738	0.9177	0.1952	0.096*	
H14B	0.4111	0.8230	0.1574	0.096*	
C13	0.5000	0.9397 (3)	0.2500	0.0588 (10)	
O3	0.43615 (17)	0.81214 (15)	0.3188 (2)	0.0711 (7)	
H3	0.4403	0.7483	0.3072	0.107*	
H13	0.5146	0.9859	0.3169	0.085*	

### Atomic displacement parameters (Å^2^)

**Table:**

	*U*^11^	*U*^22^	*U*^33^	*U*^12^	*U*^13^	*U*^23^
Ni1	0.02047 (18)	0.01993 (18)	0.02456 (19)	0.000	0.01067 (14)	0.000
S1	0.0196 (3)	0.0197 (3)	0.0300 (3)	0.000	0.0122 (2)	0.000
O1	0.0245 (6)	0.0261 (6)	0.0273 (7)	−0.0002 (5)	0.0052 (5)	0.0017 (5)
O2	0.0377 (8)	0.0369 (8)	0.0568 (10)	0.0089 (6)	0.0281 (8)	−0.0045 (7)
N1	0.0259 (7)	0.0216 (7)	0.0257 (7)	−0.0002 (6)	0.0132 (6)	−0.0002 (6)
N2	0.0236 (7)	0.0236 (7)	0.0268 (7)	0.0003 (6)	0.0120 (6)	−0.0008 (6)
C2	0.0418 (11)	0.0340 (10)	0.0274 (9)	−0.0021 (9)	0.0167 (9)	−0.0068 (8)
C6	0.0268 (9)	0.0398 (11)	0.0372 (10)	−0.0050 (8)	0.0170 (8)	0.0041 (9)
C7	0.0250 (8)	0.0324 (9)	0.0285 (9)	0.0016 (7)	0.0134 (8)	0.0045 (7)
C9	0.0417 (11)	0.0384 (11)	0.0291 (9)	0.0038 (9)	0.0205 (9)	−0.0027 (8)
C5	0.0251 (9)	0.0339 (10)	0.0342 (10)	−0.0075 (8)	0.0099 (8)	−0.0005 (8)
C4	0.0251 (8)	0.0243 (8)	0.0262 (8)	−0.0013 (7)	0.0086 (7)	0.0017 (7)
C10	0.0307 (9)	0.0288 (9)	0.0279 (9)	0.0002 (7)	0.0129 (8)	−0.0044 (7)
C8	0.0343 (10)	0.0455 (12)	0.0333 (10)	0.0005 (9)	0.0221 (9)	0.0024 (9)
C1	0.0343 (10)	0.0290 (9)	0.0304 (9)	−0.0015 (8)	0.0186 (8)	−0.0019 (7)
C11	0.0216 (8)	0.0223 (8)	0.0238 (8)	0.0016 (6)	0.0092 (7)	0.0024 (7)
C3	0.0321 (10)	0.0280 (9)	0.0276 (9)	−0.0054 (7)	0.0089 (8)	−0.0050 (7)
C12	0.0222 (8)	0.0202 (8)	0.0243 (8)	0.0004 (6)	0.0100 (7)	0.0023 (6)
C14	0.077 (2)	0.081 (2)	0.080 (2)	0.0248 (19)	0.0404 (19)	0.0057 (19)
C13	0.091 (3)	0.0280 (15)	0.070 (2)	0.000	0.051 (2)	0.000
O3	0.1204 (19)	0.0423 (10)	0.0973 (16)	0.0019 (10)	0.0895 (16)	−0.0033 (10)

### Geometric parameters (Å, °)

**Table:**

Ni1—N2^i^	2.0775 (16)	C7—C11	1.402 (3)
Ni1—N2	2.0775 (16)	C7—C8	1.406 (3)
Ni1—N1	2.0802 (16)	C9—C8	1.362 (3)
Ni1—N1^i^	2.0802 (16)	C9—C10	1.396 (3)
Ni1—O1^i^	2.1127 (14)	C9—H9A	0.9300
Ni1—O1	2.1127 (14)	C5—C4	1.431 (3)
Ni1—S1	2.6758 (9)	C5—H5A	0.9300
S1—O2^i^	1.4559 (14)	C4—C12	1.408 (2)
S1—O2	1.4559 (14)	C4—C3	1.410 (3)
S1—O1	1.4950 (14)	C10—H10A	0.9300
S1—O1^i^	1.4950 (14)	C8—H8A	0.9300
N1—C1	1.332 (2)	C1—H1A	0.9300
N1—C12	1.358 (2)	C11—C12	1.436 (2)
N2—C10	1.332 (2)	C3—H3A	0.9300
N2—C11	1.359 (2)	C14—O3	1.384 (4)
C2—C3	1.362 (3)	C14—C13	1.505 (4)
C2—C1	1.403 (3)	C14—H14A	0.9700
C2—H2A	0.9300	C14—H14B	0.9700
C6—C5	1.353 (3)	C13—C14^i^	1.505 (4)
C6—C7	1.433 (3)	C13—H13	0.9659
C6—H6A	0.9300	O3—H3	0.8200
			
N2^i^—Ni1—N2	170.54 (8)	C5—C6—H6A	119.7
N2^i^—Ni1—N1	93.90 (6)	C7—C6—H6A	119.7
N2—Ni1—N1	80.05 (6)	C11—C7—C8	116.97 (17)
N2^i^—Ni1—N1^i^	80.05 (6)	C11—C7—C6	119.45 (18)
N2—Ni1—N1^i^	93.90 (6)	C8—C7—C6	123.55 (18)
N1—Ni1—N1^i^	101.07 (9)	C8—C9—C10	119.43 (18)
N2^i^—Ni1—O1^i^	95.71 (6)	C8—C9—H9A	120.3
N2—Ni1—O1^i^	92.14 (6)	C10—C9—H9A	120.3
N1—Ni1—O1^i^	161.48 (5)	C6—C5—C4	121.57 (18)
N1^i^—Ni1—O1^i^	96.16 (6)	C6—C5—H5A	119.2
N2^i^—Ni1—O1	92.14 (6)	C4—C5—H5A	119.2
N2—Ni1—O1	95.71 (6)	C12—C4—C3	117.17 (17)
N1—Ni1—O1	96.16 (6)	C12—C4—C5	118.88 (17)
N1^i^—Ni1—O1	161.48 (5)	C3—C4—C5	123.95 (17)
O1^i^—Ni1—O1	67.73 (7)	N2—C10—C9	122.67 (18)
N2^i^—Ni1—S1	94.73 (4)	N2—C10—H10A	118.7
N2—Ni1—S1	94.73 (4)	C9—C10—H10A	118.7
N1—Ni1—S1	129.47 (4)	C9—C8—C7	119.88 (18)
N1^i^—Ni1—S1	129.47 (4)	C9—C8—H8A	120.1
O1^i^—Ni1—S1	33.86 (4)	C7—C8—H8A	120.1
O1—Ni1—S1	33.86 (4)	N1—C1—C2	122.82 (18)
O2^i^—S1—O2	110.81 (14)	N1—C1—H1A	118.6
O2^i^—S1—O1	110.68 (9)	C2—C1—H1A	118.6
O2—S1—O1	110.29 (8)	N2—C11—C7	123.16 (16)
O2^i^—S1—O1^i^	110.29 (8)	N2—C11—C12	117.02 (15)
O2—S1—O1^i^	110.68 (9)	C7—C11—C12	119.80 (16)
O1—S1—O1^i^	103.90 (11)	C2—C3—C4	119.49 (18)
O2^i^—S1—Ni1	124.60 (7)	C2—C3—H3A	120.3
O2—S1—Ni1	124.60 (7)	C4—C3—H3A	120.3
O1—S1—Ni1	51.95 (5)	N1—C12—C4	123.17 (17)
O1^i^—S1—Ni1	51.95 (5)	N1—C12—C11	117.15 (15)
S1—O1—Ni1	94.19 (7)	C4—C12—C11	119.66 (16)
C1—N1—C12	117.76 (16)	O3—C14—C13	112.9 (3)
C1—N1—Ni1	129.29 (13)	O3—C14—H14A	109.0
C12—N1—Ni1	112.78 (12)	C13—C14—H14A	109.0
C10—N2—C11	117.89 (16)	O3—C14—H14B	109.0
C10—N2—Ni1	129.14 (13)	C13—C14—H14B	109.0
C11—N2—Ni1	112.88 (12)	H14A—C14—H14B	107.8
C3—C2—C1	119.57 (18)	C14—C13—C14^i^	111.4 (3)
C3—C2—H2A	120.2	C14—O3—H3	109.5
C1—C2—H2A	120.2	C14—O3—H13	51.3
C5—C6—C7	120.55 (18)	H3—O3—H13	134.4
			
N2^i^—Ni1—S1—O2^i^	−3.49 (9)	O1—Ni1—N2—C10	−85.94 (16)
N2—Ni1—S1—O2^i^	176.51 (9)	S1—Ni1—N2—C10	−51.94 (16)
N1—Ni1—S1—O2^i^	−102.49 (10)	N1—Ni1—N2—C11	2.48 (12)
N1^i^—Ni1—S1—O2^i^	77.51 (10)	N1^i^—Ni1—N2—C11	−98.09 (12)
O1^i^—Ni1—S1—O2^i^	89.72 (10)	O1^i^—Ni1—N2—C11	165.58 (12)
O1—Ni1—S1—O2^i^	−90.28 (10)	O1—Ni1—N2—C11	97.75 (12)
N2^i^—Ni1—S1—O2	176.51 (9)	S1—Ni1—N2—C11	131.75 (11)
N2—Ni1—S1—O2	−3.49 (9)	C5—C6—C7—C11	−1.5 (3)
N1—Ni1—S1—O2	77.51 (10)	C5—C6—C7—C8	176.34 (19)
N1^i^—Ni1—S1—O2	−102.49 (10)	C7—C6—C5—C4	2.1 (3)
O1^i^—Ni1—S1—O2	−90.28 (10)	C6—C5—C4—C12	0.0 (3)
O1—Ni1—S1—O2	89.72 (10)	C6—C5—C4—C3	−179.52 (19)
N2^i^—Ni1—S1—O1	86.79 (8)	C11—N2—C10—C9	−0.7 (3)
N2—Ni1—S1—O1	−93.21 (8)	Ni1—N2—C10—C9	−176.81 (14)
N1—Ni1—S1—O1	−12.21 (8)	C8—C9—C10—N2	0.0 (3)
N1^i^—Ni1—S1—O1	167.79 (8)	C10—C9—C8—C7	0.1 (3)
O1^i^—Ni1—S1—O1	180.0	C11—C7—C8—C9	0.3 (3)
N2^i^—Ni1—S1—O1^i^	−93.21 (8)	C6—C7—C8—C9	−177.63 (19)
N2—Ni1—S1—O1^i^	86.79 (8)	C12—N1—C1—C2	−0.6 (3)
N1—Ni1—S1—O1^i^	167.79 (8)	Ni1—N1—C1—C2	−175.62 (14)
N1^i^—Ni1—S1—O1^i^	−12.21 (8)	C3—C2—C1—N1	−0.1 (3)
O1—Ni1—S1—O1^i^	180.0	C10—N2—C11—C7	1.2 (3)
O2^i^—S1—O1—Ni1	118.37 (8)	Ni1—N2—C11—C7	177.92 (13)
O2—S1—O1—Ni1	−118.64 (8)	C10—N2—C11—C12	179.30 (15)
O1^i^—S1—O1—Ni1	0.0	Ni1—N2—C11—C12	−3.94 (19)
N2^i^—Ni1—O1—S1	−95.30 (7)	C8—C7—C11—N2	−1.0 (3)
N2—Ni1—O1—S1	89.97 (7)	C6—C7—C11—N2	177.04 (17)
N1—Ni1—O1—S1	170.54 (6)	C8—C7—C11—C12	−179.08 (16)
N1^i^—Ni1—O1—S1	−31.0 (2)	C6—C7—C11—C12	−1.1 (3)
O1^i^—Ni1—O1—S1	0.0	C1—C2—C3—C4	0.0 (3)
N2^i^—Ni1—N1—C1	−12.76 (16)	C12—C4—C3—C2	0.8 (3)
N2—Ni1—N1—C1	174.57 (17)	C5—C4—C3—C2	−179.67 (19)
N1^i^—Ni1—N1—C1	−93.37 (16)	C1—N1—C12—C4	1.5 (3)
O1^i^—Ni1—N1—C1	108.4 (2)	Ni1—N1—C12—C4	177.32 (13)
O1—Ni1—N1—C1	79.82 (16)	C1—N1—C12—C11	−177.07 (16)
S1—Ni1—N1—C1	86.63 (16)	Ni1—N1—C12—C11	−1.27 (18)
N2^i^—Ni1—N1—C12	172.05 (12)	C3—C4—C12—N1	−1.6 (3)
N2—Ni1—N1—C12	−0.63 (11)	C5—C4—C12—N1	178.81 (16)
N1^i^—Ni1—N1—C12	91.44 (12)	C3—C4—C12—C11	176.96 (16)
O1^i^—Ni1—N1—C12	−66.8 (2)	C5—C4—C12—C11	−2.6 (2)
O1—Ni1—N1—C12	−95.37 (12)	N2—C11—C12—N1	3.6 (2)
S1—Ni1—N1—C12	−88.56 (12)	C7—C11—C12—N1	−178.23 (15)
N1—Ni1—N2—C10	178.78 (17)	N2—C11—C12—C4	−175.08 (15)
N1^i^—Ni1—N2—C10	78.21 (16)	C7—C11—C12—C4	3.1 (2)
O1^i^—Ni1—N2—C10	−18.11 (16)	O3—C14—C13—C14^i^	65.6 (2)

Symmetry codes: (i) −*x*+1, *y*, −*z*+1/2.

### Hydrogen-bond geometry (Å, °)

**Table:**

*D*—H···*A*	*D*—H	H···*A*	*D*···*A*	*D*—H···*A*
O3—H3···O2i	0.82	1.92	2.743 (2)	179

Symmetry codes: i.
